# Complex management and descriptive cost analysis of kidney transplant candidates: a descriptive cross-sectional study

**DOI:** 10.1186/s12913-024-11200-y

**Published:** 2024-06-24

**Authors:** Guillermo Pedreira-Robles, Paloma Garcimartín, María José Pérez-Sáez, Anna Bach-Pascual, Marta Crespo, Victoria Morín-Fraile

**Affiliations:** 1 Nephrology Department, Hospital del Mar, Parc de Salut Mar, Barcelona, Spain; 2 ESIMar (Mar Nursing School), Parc de Salut Mar, Universitat Pompeu Fabra Affiliated, Barcelona, Spain; 3 SDHEd (Social Determinants and Health Education Research Group), IMIM (Hospital del Mar Medical Research Institute), Barcelona, Spain; 4 Nursing and Health PhD Programme, University of Barcelona, Barcelona, Spain; 5 Nursing department, Hospital del Mar, Parc de Salut Mar, Passeig Marítim 25-29, Barcelona, 08003 Spain; 6 Research Group in Nursing Care, IMIM (Hospital del Mar Medical Research Institute), Barcelona, Spain; 7 Biomedical Network Research Center for Cardiovascular Diseases, (CIBERCV, Carlos III Health Institute), Madrid, Spain; 8 Kidney Research Grup (GREN), Hospital del Mar Medical Research Institute (IMIM), RD16/0009/0013 (ISCIII FEDER REDinREN), Barcelona, Spain; 9 Department of Public Health, Mental Health, and Maternal and Child Health, Faculty of Nursing, University of Barcelona, Barcelona, Spain

**Keywords:** Care, Complexity, Health economics, Kidney transplant, Management, Nursing

## Abstract

**Background:**

The organisational care needs involved in accessing kidney transplant have not been described in the literature and therefore a detailed analysis thereof could help to establish a framework (including appropriate timing, investment, and costs) for the management of this population. The main objective of this study is to analyse the profile and care needs of kidney transplant candidates in a tertiary hospital and the direct costs of studying them.

**Methods:**

A descriptive, cross-sectional study was conducted using data on a range of variables (sociodemographic and clinical characteristics, study duration, and investment in visits and supplementary tests) from 489 kidney transplant candidates evaluated in 2020.

**Results:**

The comorbidity index was high (> 4 in 64.3%), with a mean of 5.6 ± 2.4. Part of the study population had certain characteristics that could hinder their access a kidney transplant: physical dependence (9.4%), emotional distress (33.5%), non-adherent behaviours (25.2%), or language barriers (9.4%). The median study duration was 6.6[3.4;14] months. The ratio of required visits to patients was 5.97:1, meaning an investment of €237.10 per patient, and the ratio of supplementary tests to patients was 3.5:1, meaning an investment of €402.96 per patient.

**Conclusions:**

The study population can be characterised as complex due to their profile and their investment in terms of time, visits, supplementary tests, and direct costs. Management based on our results involves designing work-adaptation strategies to the needs of the study population, which can lead to increased patient satisfaction, shorter waiting times, and reduced costs.

## Background

The care of individuals with complex care needs has been extensively addressed in care management strategies, concluding that individuals who seek care from the health system and have certain characteristics that identify them as complex consume between 40 and 75% of available resources [1, 2]. These studies on the general adult population over 65 years of age estimate the complexity prevalence to be around 3% to 5%, defining it as the difficulty in managing a person’s care and the need to implement specific individual plans due to the presence or concurrence of illnesses, as well as their way of using services or the characteristics of their environment [1].

Against this backdrop, chronic kidney disease is a public health problem with an increasing prevalence [3, 4]. In addition, the most recent epidemiological studies conclude that individuals with chronic kidney disease are frequently part of the ageing population and have more comorbidities in addition to chronic kidney disease itself [4–6]. As a result, this population has generally been described in several ways: complex, comorbid, multi-pathological, fragile, etc. Due to their increased risk of complications [6, 7] and also the increased health costs involved in their care [8, 9], this group has unique characteristics that warrant specialised care. A thorough assessment of the kidney transplant candidate is therefore essential to reduce the risks associated with the transplant process [10]. Furthermore, this assessment may now require more investment, in all respects, given the ageing profile of the current population [9, 11].

Despite these considerations, kidney transplant remains the best treatment option for the vast majority of individuals with chronic kidney disease in terms of quality of life, survival, and costs [12–14], with no upper age limit identified for kidney transplant [4, 10]. There is, however, an estimated increased risk of complications and postoperative mortality in those who may meet criteria for frailty and comorbidity [6, 15], which, as reported, are increasingly prevalent with age. If a living-donor kidney transplant is not an option, it is difficult to predict when the opportunity to receive a kidney transplant may come, with estimated waiting times, in our setting, at a median of 24 months from the start of dialysis [4], During this waiting period, close monitoring of the kidney transplant candidate by the professionals involved will be required, depending on the needs of the individual, to confirm that this treatment option is still feasible and safe [4, 10].

The organisational care needs involved in accessing kidney transplant have not been described in the literature and therefore a detailed analysis thereof could help to establish a framework (including appropriate timing, investment, and costs) for the management of this complex population. The primary objective of this study is to analyse the profile of kidney transplant candidates and their care needs in a tertiary hospital as well as the direct costs of studying them in order to assess the complexity of managing this process. This analysis will allow us to propose more efficient strategies for kidney transplant candidates and for the health system as a whole.

## Methods

### Design

A cross-sectional analysis was carried out on all kidney transplant candidates who were being monitored in the kidney transplant access consultation as of 31 December 2020. This research adheres to the STROBE (Strengthening the Reporting of Observational studies in Epidemiology) guidelines for the reporting of observational studies [16].

### Setting

The care process that includes kidney transplant access in our benchmark healthcare facility is made up of 3 monographic nursing, nephrology, and urology consultations. In line with the recommendations set out in the KDIGO guidelines [10], these unit specialists are directly involved in the assessment of kidney transplant candidates. In addition, there are monographic cardiology, pulmonology, and anaesthesiology consultations for referring and/or examining kidney transplant candidates that require specialised assessments, and other general consultations typical of a tertiary hospital, for professional opinion in relation to the case and according to the person’s needs.

The assessments made by the various professionals are accompanied by the necessary supplementary tests and examinations to weigh up the risks and benefits of the requested procedure. If these assessments are favourable, the person is deemed eligible for kidney transplant as a treatment option – without being able to predict exactly when the opportunity to receive such treatment will come, unless there is the option of a living-donor kidney transplant, which would allow the procedure to be scheduled. During the time on the kidney transplant waiting list, the kidney transplant candidate is assessed annually by the transplant facility team, so that this treatment option can continue to be considered as a viable and safe choice with the support of the teams from the benchmark healthcare facilities during follow-up. In addition, the necessary supplementary tests and examinations are updated periodically based on the service’s action protocol and the individual’s clinical progress and needs.

Candidates may arrive at the kidney transplant access pathway already having had the supplementary tests done at the healthcare facility of origin or they may need to undergo a full work-up at the referral transplant facility. At the time of this study analysis, regardless of the kidney transplant access model, kidney transplant candidates had the same number of visits, and the study duration was similar. The tests included in this study are those performed by the referral transplant facility and do not include other external tests that may be part of the kidney transplant candidate assessment process.

For the purpose of this article, an ‘uneventful study’ of a kidney transplant candidate has been defined as one requiring an initial assessment consultation, completion of the necessary supplementary tests, and a second assessment before a decision can be made to include the person in the kidney transplant programme. Due to various circumstances, cases where not all of the necessary information was available at the time of the second consultation were labelled as ‘study with incidents.’ Examining the causes of these incidents in relation to the care needs of the study population will enable us to gain a deeper understanding of the care pathways involved and make recommendations for improving their care.

In relation to costs, it is worth noting that, within the study context, costs are always funded by the public healthcare system, which is financed through direct taxes on the citizenry.

### Sample

The study focuses on kidney transplant candidates who were being monitored in the kidney transplant access consultation as of 31 December 2020, thereby indicating that the costs pertain to the year 2020. Patients who had not completed the study as kidney transplant candidates or who had not attended the final consultation to be assessed for inclusion in the kidney transplant programme were excluded.

### Data collection

The following variables were collected from the electronic health records at the referral facility:

- Sociodemographic variables: sex; age; employment status; place of birth; place of residence; religion; and language barriers.
- Clinical variables: renal replacement therapy; dependency in activities of daily living (Barthel index < 90); emotional distress during treatment as documented in the clinical record; non-adherent behaviours as documented in the clinical record; Charlson Comorbidity Index; high blood pressure; dyslipidaemia; diabetes mellitus; body mass index; physical activity of less than 30 min per day; tobacco use; chronic obstructive pulmonary disease and/or asthma; coronary artery disease.
- Study duration and implications: study duration; reasons for incidents during the study.
- Number of visits, types of visits (in-person or remote), and costs of the visits required in 2020 as part of the assessment.
- Number, type, and direct costs of supplementary tests and examinations carried out in 2020.

These variables were agreed upon by the members of the research team and after a thorough review of the relevant literature published in current clinical practice guidelines [10]. The Charlson Comorbidity Index score has been shown to be linked to potential complications among kidney transplant candidates [17], as well as to having a body mass index score in the obese range [18]. Similarly, cardiovascular risk factors are highly prevalent in this population, meaning increased costs when studying kidney transplant candidates [19, 20].

In the financial assessment, only variables within the analysis facility were quantified, without considering indirect costs or costs associated with other facilities. The cost of each activity analysed is based on the cost tables of the referral facility.

### Data analysis

The results were recorded and analysed using a database created by the research team using SPSS© (version 26) from IBM (IBM Corporation). A descriptive analysis of the study variables was carried out: qualitative variables were expressed as absolute and relative frequencies, while quantitative variables were expressed as means and standard deviations in the case of a normal distribution and as medians and interquartile ranges in the case of a non-normal distribution. For the economic analysis, the ratio of visits or costs per patient was used.

## Results

**Fig. 1:**
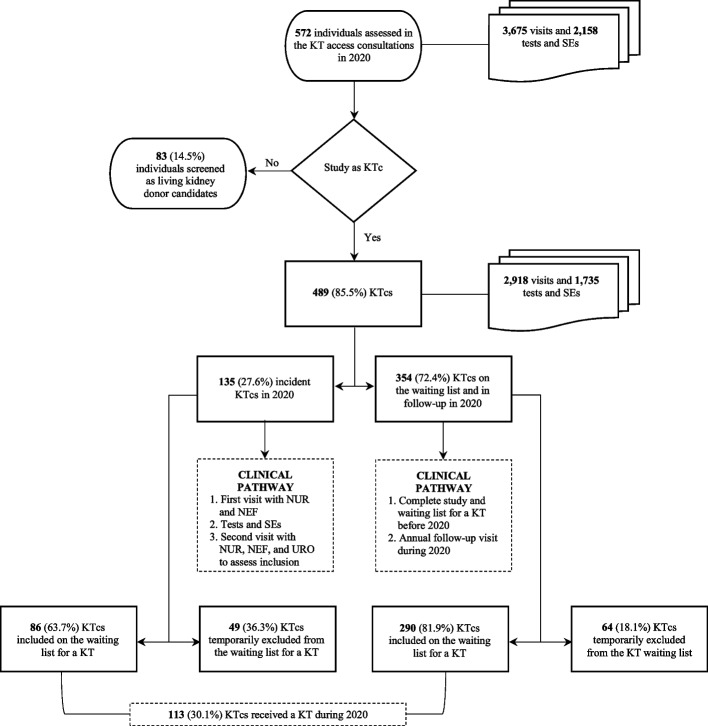
Flow diagram showing the participant inclusion process. Note: KT, kidney transplant; KTc, kidney transplant candidate; SEs, supplementary examinations; NUR, Nursing; NEF, Nephrology; URO, Urology

The sample selection and care pathways are depicted in Fig. 1. This flow diagram illustrates the participant inclusion process. In 2020, a total of 489 individuals were studied as candidates for kidney transplantation. This sample represents 85.5% of the individuals (recipient candidates and living kidney donors) assessed in the kidney transplant access consultations during the study period. Of these, 135 (27.6%) were incident patients in the evaluation process throughout the year, and 86 (63.7%) were placed on the waiting list for a kidney transplant. 354 (72.4%) candidates were either on the waiting list and under follow-up (*n* = 290; 81.9%) or temporarily excluded and under follow-up (*n* = 64; 18.1%). A total of 113 candidates underwent kidney transplantation during 2020.

### Analysis of sociodemographic and clinical characteristics

Table 1 shows the sociodemographic and clinical characteristics of the sample analysed.

**Table 1:** Sociodemographic and clinical characteristics of the kidney transplant candidates studied (*n* = 489)

**Sociodemographic characteristics**	
**Sex (n; %)**	
Female	147 (30.1%)
Male	342 (69.9%)
**Age (mean; SD)** [*years*]	60.8 ± 12.5
**Employed (n; %)**	51 (10.4%)
**Place of birth (n; %)**	
Spain	374 (76.5%)
Outside Spain	115 (23.5%)
Europe	9 (1.8%)
Southeast Asia	18 (3.7%)
East Asia	11 (2.2%)
North Africa	26 (5.3%)
Sub-Saharan Africa	16 (3.3%)
Latin America	35 (7.2%)
**Place of residence (n; %)**	
City of Barcelona	159 (32.5%)
Province of Barcelona	280 (57.2%)
Rest of Catalonia	40 (8.9%)
Rest of Spain	10 (2%)
**Religion (n; %)**	
Not reported	368 (75.3%)
Islam	57 (11.7%)
Christianity	47 (9.6%)
Jehovah’s Witnesses	12 (2.5%)
Taoism	3 (0.6%)
Judaism	1 (0.2%)
Sikhism	1 (0.2%)
**Total language barrier (n; %)**	46 (9.4%)
***Clinical characteristics***	
** RRT option at the first visit (n; %)**	
ACKD pre-RRT	146 (29.9%)
HD	290 (59.3%)
PD	53 (10.8%)
**Dependency in ADL (Barthel < 90) (n; %)**	46 (9.4%)
**Diagnosis of emotional distress under treatment (n; %)**	164 (33.5%)
**Non-adherent behaviours during the study process (n; %)**	123 (25.2%)
**Charlson Comorbidity Index (mean; SD)**	5.6 ± 2.4
Score: 0–1 (n; %)	0
Score: 2 (n; %)	57 (11.7%)
Score: 3–4 (n; %)	117 (23.9%)
Score: 5–6 (n; %)	133 (27.2%)
Score: 7–8 (n; %)	123 (25.2%)
Score ≥ 9 (n; %)	59 (12.1%)
**Cardiovascular risk factors (mean; SD)**	3.4 ± 1.9
High blood pressure (n; %)	465 (95.1%)
Dyslipidaemia (n; %)	336 (68.7%)
Diabetes mellitus (n; %)	214 (43.8%)
BMI (mean; SD) [*Kg/m*^*2*^]	27.8 ± 5.4
BMI > 29.99 (n; %) [*Kg/m*^*2*^]	156 (31.9%)
Sedentary lifestyle (n; %)	366 (74.8%)
Active tobacco use (n; %)	109 (22.3%)
Chronic obstructive pulmonary disease and/or asthma (n; %)	130 (26.6%)
Coronary artery disease (n; %)	102 (20.9%)

*ACKD* Advanced chronic kidney disease*, ADL* Activities of daily living*, BMI* Body mass index*, HD* Haemodialysis*, PD* Peritoneal dialysis*, RRT* Renal replacement treatment*, SD* Standard deviation

Part of the study population exhibited certain characteristics that may have implications for the outcome of the kidney transplant, such as: physical dependence (9.4%), emotional distress during treatment (33.5%), non-adherent behaviours during the study process that resulted in missed visits and/or missed supplementary tests (25.2%), and language barriers (9.4%).

**Fig. 2:**
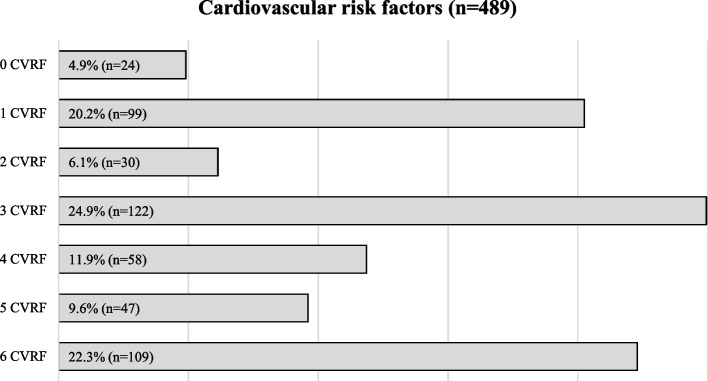
Percentage of kidney transplant candidates studied with cardiovascular risk factors. Note: CVRFs included: high blood pressure; dyslipidaemia; diabetes mellitus; obesity; sedentary lifestyle; and smoking habit (see Table 1)

The comorbidity index was high (> 4 in 64.3%), with a mean of 5.6 ± 2.4 (Table 1) and the study cardiovascular risk factors were prevalent in our sample (> 3 in 68.7%), with a mean of 3.4 ± 1.9 cardiovascular risk factors (Fig. 2).

In the patients classified as ‘uneventful’, the mean comorbidity index was 5 ± 2, while in the group of patients classified as ‘with incidents’ the mean comorbidity index was 6 ± 2.4.

### Study duration and implications

Table 2 shows the study duration and its implications for the study of kidney transplant candidates.

**Table 2:** Duration and implications for studies of kidney transplant candidates analysed during 2020 (*n* = 489)

	**Uneventful studies (n; %)**	**Studies with incidents (n; %)**	**Total (n; %)**
**Sample (n; %)**	179 (36.6%)	310 (63.4%)	489 (100%)
**Study duration (median; IQR)** [*months*]	2.9 [1.8; 4.3]	11.7 [7.2; 20]	6.6 [3.4; 14]
**Reasons for incidents (*n***=**580)** **(n; %)**			
Extending the assessment with cardiology tests and visits (n; %)	**-**	98 (16.9%)	**-**
GFR > 15 ml/min, precluding inclusion in the WL (n; %)	**-**	75 (12.9%)	**-**
Unjustified missed visits or STs / Non-adherence (n; %)	**-**	70 (12.1%)	**-**
The COVID-19 pandemic (n; %)	**-**	37 (6.4%)	**-**
Justified missed visits or STs due to hospital admission (n; %)	**-**	33 (5.7%)	**-**
Identification of acute decompensated disease (n; %)	**-**	28 (4.8%)	**-**
Extending the study with gastroenterological tests and visits (n; %)	**-**	24 (4.1%)	**-**
Extending the study with neurological tests and visits (n; %)	**-**	21 (3.6%)	**-**
Extending the study with urological tests and visits (n; %)	**-**	20 (3.5%)	**-**
Extending the study with angiology tests and visits (n; %)	**-**	17 (2.9%)	**-**
Potential LDKT (n; %)	**-**	16 (2.8%)	**-**
Clinical pathway without KT access nurse (n; %)	**-**	16 (2.8%)	**-**
Active abuse of illegal drugs (n; %)	**-**	7 (1.2%)	**-**

*STs* Supplementary tests, *GFR* Glomerular filtration, *IQR* Interquartile range, *KT* Kidney transplant, *LDKT* Living-donor kidney transplant, *WL* Waiting list

Patients categorised as ‘uneventful’ accounted for 36.6% of the total, and 65.8% (*n* = 204) of the patients classified as ‘with incidents’ (*n* = 310; 63.4%) had a single cause for those incidents, while 34.2% (*n* = 106) had more than one cause. The main causes in the patients analysed were: the need to extend the assessment with cardiology tests and visits (16.9%); the need to extend the study with another medical or surgical speciality (14.1%); having a glomerular filtration above 15 ml/min (12.9%); and non-adherence as evidenced by unjustified missed visits and/or examinations (12.1%).

### Visits made and costs

Table 3 presents the type, format, and financial costs of the 2,918 visits required in the study of the 489 kidney transplant candidates analysed. These visits account for 79.4% of the total annual activities carried out in the kidney transplant access consultations, with the remainder corresponding to the study of living kidney donor candidates. The ratio of required visits to patients was 5.97:1, representing an investment of €237.10 per patient.

**Table 3:** Visits made with KT candidates (*n* = 489) and costs in 2020

Type of visit	Average visits per patient (ratio visit/patient)	Average costs per patient
**Nursing visits**	**2.66**	**€96.82**
Face-to-face	1.02	€44.06
Non-face-to-face	1.59	€52.76
**Nephrology visits**	**1.63**	**€62.21**
Face-to-face	0.59	€27.74
Non-face-to-face	1.04	€34.47
**Urology visits**	**0.35**	**€19.85**
Face-to-face	0.31	€18.49
Non-face-to-face	0.04	€1.36
**Psychology visits**	**0.78**	**€31.22**
Face-to-face	0.18	€11.3
Non-face-to-face	0.6	€19.92
**Cardiology visits**	**0.19**	**€10.36**
Face-to-face	0.14	€8.86
Non-face-to-face	0.04	€1.5
**Neurology visits**	**0.11**	**€6.1**
Face-to-face	0.08	€4.88
Non-face-to-face	0.04	€1.22
**Infectious Diseases visits**	**0.07**	**€3.69**
Face-to-face	0.05	€3.21
Non-face-to-face	0.01	€0.48
**Anaesthesia visits**	**0.05**	**€2.36**
Face-to-face	0.02	€1.54
Non-face-to-face	0.02	€0.82
**General/Plastic Surgery face-to-face visits**	**0.01**	**€0.39**
**Interdisciplinary Committee on KT non-face-to-face visits**	**0.12**	**€4.08**
**TOTAL**	**5.97**	**€237.1**

*KT* Kidney transplant

### Supplementary tests and examinations carried out and costs

Table 4 shows the type, format, and financial costs of the 1,735 supplementary tests and examinations necessary for studying the 489 kidney transplant candidates analysed. These supplementary tests and examinations account for 80.4% of the total annual activities carried out in the kidney transplant access consultations, with the remainder corresponding to the study of living kidney donor candidates. The ratio of required supplementary tests to patients was 3.5:1, representing an investment of €402.96 per patient.

**Table 4:** Supplementary tests performed on kidney transplant candidates (*n* = 489) and costs in 2020

Type of test	Average tests per patient (ratio test/patient)	Average costs per patient
Abdominal ultrasound	0.6	€10.29
Chest X-ray	0.46	€2.8
Abdominal computed tomography angiogram	0.46	€122.86
Analytics	0.38	€149.94
Echocardiogram	0.31	€51.82
Myocardial ischaemia test	0.27	€2.09
Faecal occult blood test	0.25	€1.03
Respiratory function tests	0.21	€18.64
Prostate-specific antigen (PSA) test^a^	0.2^a^	€0
Computed tomography scan of the chest	0.11	€9.22
Electrocardiogram	0.11	€1.05
Crossmatch test	0.08	€0
Cranial computed tomography angiogram	0.06	€3.75
Mammogram^b^	0.17^b^	€0.64
Transoesophageal ultrasound	0.02	€2.76
Elastography	0.02	€2.17
Colonoscopy	0.02	€2.08
Cytology^b^	0.03^b^	€0.4
Magnetic resonance imaging of the prostate^a^	0.01^a^	€0.98
Chest ultrasound	0.004	€0.07
Magnetic resonance imaging of the heart	0.002	€0.12
Polysomnography	0.002	€0.72
**TOTAL (n; %)**	**3.5**	**€402.96**

^a^Only in men

^b^Only in women

## Discussion

This study is the first to report on the current profile of kidney transplant candidates in a tertiary hospital in order to assess the complexity of managing this group and the direct costs that can be incurred. Our analysis suggests that the current profile of kidney transplant candidates has changed over the past two decades, with an increase in the clinical and management complexity of this population. Our analysis has also revealed a great number of associated comorbidities and a high financial investment required for the current study of kidney transplant candidates.

In the study population, comorbidity (as measured with the Charlson comorbidity index) and associated cardiovascular risk factors are prevalent, which is similar to that reported in current registers in our setting [4, 21] and in other international contexts [22, 23]. However, the current profile differs substantially from that reported in the literature of the 1990s and 2000s [24]. Currently, individuals with high comorbidity indices are accepted as kidney transplant candidates due to the evidence-based benefits of this treatment option as compared with other therapeutic strategies [10]. This treatment option has been reported to improve the individual’s chance of survival [25] and quality of life [26], having a positive impact on the health system as a whole, [27] provided that these comorbidities are studied and monitored by the specialists in the interdisciplinary referral team [10, 14]. It is therefore safe to say that the clinical profile of kidney transplant candidates today displays higher levels of comorbidity than in past decades, which can lead to greater complexity in their care.

We are aware that the current profile of kidney transplant candidates is also associated with an increased risk of cardiovascular events, malignancy, and/or potential complications compared to the general population [9, 28–30], which can also determine whether the individual is unsuitable for a kidney transplant [27]. In our results, we find that more than 35% of the individuals studied de novo could not be included on the waiting list for a kidney transplant due to issues identified during the study or incompatibilities in accessing this treatment option. In addition, more than 15% remain off the waiting list more than one year after this initial study. These data suggest that, in general, the study population has complex characteristics relating to the management of their disease process.

Other characteristics found in our study have also been reported in the literature that individually assesses the complexity of managing the process and its relationship to socio-economic and cultural factors [31]. The literature explains that these are characteristics linked to socio-economic vulnerability, such as being a foreign national, language limitations, little social support, low income, and low level of education [31–33], which may delay or even prevent access to this treatment option.

Considering the profile of comorbidities and the socio-economic limitations of the population examined, our study reported more than 60% of kidney transplant access processes as having incidents. These cases with incidents take a long time (> 11 months) before they can access the kidney transplant programme due to the higher complexity of their process management. Most of the delays in the kidney transplant access process are caused by the need for further kidney transplant candidate assessment by another medical speciality and by non-adherent behaviours involving missed visits or supplementary tests. In the literature, non-adherence figures of 52% to 67% have been reported in the population who end up receiving kidney transplant [34], pointing to a significantly greater problem than that reported in our study.

Other causes of delay in the study, which make the management of the process even more complex, include missing visits or supplementary tests due to hospital admissions, detecting acute decompensated disease, and active abuse of illegal drugs. A study conducted with the same population reported that 38% of kidney transplant candidates were admitted to hospital at least once during their first year on the waiting list, rising to almost 50% in cases in which the individual met frailty criteria [35]. In addition, glomerular filtration levels far above 15 ml/min and the consequences of the COVID-19 pandemic, among others, call for a prior analysis of both the kidney transplant candidate’s situation and the hospital setting to avoid starting the study in circumstances that could prevent the kidney transplant candidate from being included in the kidney transplant programme. Taking into consideration the causes that may lead to study delays, we have detailed the benefits of having a kidney transplant access nurse to carry out a planning and optimisation analysis before and during the study process, as well as setting up a care plan to achieve the desired objectives before the surgical procedure takes place [36].

Despite the clinical and management complexity found in the study population, the care strategy to be followed is to study, and consequently minimise, the characteristics and risks through an interdisciplinary plan and thus continue to offer kidney transplant as the best treatment option for individuals with chronic kidney disease [10, 12–14, 17, 35]. It is therefore essential to have management models in place that include effective strategies for establishing the candidate’s profile and determining differentiated strategies for the most complex cases – reducing waiting times and speeding up visits and supplementary examinations in the study of this population [36].

When considering the direct annual investment involved in a kidney transplant access programme in a tertiary hospital (in terms of tests, visits, and costs), we reported in a previous study conducted in the same context that, despite the greater clinical complexity of kidney transplant candidates, the study of kidney donor candidates requires twice the number of visits and supplementary tests in a shorter period of time [37]. A distinction must be drawn between clinical or physical complexity and the complexity involved in the care management of a healthcare process. In this case, it can be concluded that the study of kidney donor candidates is more complex to manage, despite the fact that the clinical complexity is lower [38].

Compared to previous studies, the overall cost of a kidney transplant is lower than that reported for dialysis therapies [14, 39], which sufficiently justifies the reported investment and the strategies that could be implemented to increase the efficiency of this treatment option. In the study country (Spain), highly variable mean costs per disease have been reported, as costs increase with the number of co-occurring diseases. In general, an individual with one comorbidity amounts to a mean of €413 per year; an individual with five comorbidities, €2,413 per year; and an individual with ten comorbidities, €9,626 per year [40]. This study has only analysed the direct costs involved in accessing kidney transplant in a transplant facility, irrespective of previous studies carried out in the referral facility or the need to attend to other intercurrent care processes. A suitability study for a kidney transplant in our setting costs a mean of €640.06 per patient.

Taking the profile of kidney transplant candidates and their mean healthcare costs into account, it is safe to say that we are facing a care framework that is not just complex in terms of disease, but also in terms of management. The majority of the studies in this area of analysis conclude that heavy care workloads are one of the most important factors that most negatively impacts professionals and their role in relation to the individuals they care for [41, 42]. The association between patient outcomes and staff outcomes has generated evidence in two respects: the needs of patients and the needs of professionals [42].

In relation to the needs of professionals, one of the main sources of professional stress is the high workload that can result from providing care directly to highly complex patients [43]. Professional stress is closely related to emotional exhaustion, depersonalisation, and low professional accomplishment, all of which can lead to burnout syndrome [44]. These heavy care workloads have a direct negative impact not only on the professionals, but also on the quality of the care they provide, which is poorer in settings with heavier workloads [45]. This takes us into the field of patients’ needs, which are closely related to their caregivers’ needs and are becoming ever more specific due to the aforementioned complexities. It has thus been established that adequate staffing is essential to high-quality care, satisfaction with the care received, and shorter waiting times in populations defined as complex [46, 47].

### Limitations of research

The primary limitation of this study lies in its planned single-centre design and the selective inclusion of variables, which solely address clinical and direct cost issues at a specific moment in the kidney transplant pathway. However, while this analysis can be replicated in other contexts to gather further evidence on the study objective, it's imperative to acknowledge the absence of indirect costs in our assessment, as well as the broader implications overlooked by focusing solely on this moment of the evaluation process. These omissions restrict our ability to comprehensively evaluate the economic impact on the healthcare system and the overall burden borne by kidney transplant candidates. By solely focusing on variables associated with direct costs within the transplant process at the referral facility, we provide clarity on this aspect but inadvertently overlook the broader financial implications and the comprehensive needs of the target population.

On the other hand, it is important to note that this study collects data from the year 2020, which was marked by the COVID-19 pandemic. During the months of lockdown and heightened severity (March and April), the kidney transplant access unit at the studied centre was closed, but all lost activity was promptly resumed thereafter.

### Implications clinical practice

When comparing the results reported in this study with the reference literature, it seems vital that agreement is reached on the best organisational and management strategies to cater for this population with complex care needs. The involvement of nurses in providing access to kidney transplant has been reported to be beneficial in this regard [36], but is not yet widespread in our study context [38]. Therefore, it is necessary to continue to provide robust evidence of these benefits and to engage managers and policy makers to bring greater quality, effectiveness, and efficiency to the kidney transplant treatment option.

The first of these measures that we have adopted in our referral facility is an initial virtual appointment via a phone call to the kidney transplant candidate to review their clinical record, the evidence they provide, and their needs, to be able to plan the best organisational strategy for them and the people around them. This strategy aims to avoid initiating kidney transplant candidate evaluation at times when they may not be suitable and to prioritise the processes that will benefit from kidney transplant candidate evaluation being started. Clear examples include: avoiding starting a kidney transplant candidate study when the patient has very high glomerular filtration rates and slow clinical progress; in the context of a pandemic or other circumstances specific to the organisation of the facility; or in personal or organisational situations suggesting that it may not be the right time to initiate the study. Priority is thus given to evaluation of kidney transplant candidates who are eligible for a pre-emptive kidney transplant before starting renal replacement therapy, either because of the recipient’s own characteristics or because of the possibility of proceeding to a kidney transplant from a living donor. The implementation of this new model will be analysed to report on its efficacy and efficiency.

## Conclusions

This study provides a detailed analysis of the profile of kidney transplant candidates and their care needs within a tertiary hospital, along with an examination of the direct costs associated with their evaluation. The study population can be characterised as complex due to their profile and their investment in terms of time, visits, supplementary tests, and direct costs. It illuminates the clinical complexity of this population and underscores the need for comprehensive interdisciplinary care planning to address their evolving needs. Management based on our results involves designing work-adaptation strategies tailored to the needs of the study population, which can lead to increased patient satisfaction, shorter waiting times, and reduced costs. Despite advances in the current management of this population, challenges persist, leading to delays or limitations in access to transplant programs and contributing to the complexity of treatment.

## Data Availability

The datasets used and/or analysed during the current study are available from the corresponding author on reasonable request.
